# Controllable Doping of Mn into Ni_0.075‐x_Mn_x_Al_0.025_(OH)_2_(CO_3_)_0.0125_·yH_2_O for Efficient Adsorption of Fluoride Ions

**DOI:** 10.1002/gch2.202300018

**Published:** 2023-04-06

**Authors:** Ararso N. Wagassa, Lemma T. Tufa, Jaebeom Lee, Enyew A. Zereffa, Tofik A. Shifa

**Affiliations:** ^1^ Department of Applied Chemistry Adama Science and Technology University P.O. Box 1888 Adama Ethiopia; ^2^ Institute of Material Chemistry Chungnam National University Deajeon 34134 South Korea; ^3^ Department of Chemistry Chungnam National University Deajeon 34134 South Korea; ^4^ Department of Molecular Science and Nanosystem Ca’ Foscari University of Venice Via Torino 155 Venezia Mestre 30172 Italy

**Keywords:** adsorption, aqueous fluoride ions, characterization, layered double hydroxides

## Abstract

Here, the structural, optical, and adsorptive behaviors of Ni_0.075‐x_Mn_x_Al_0.025_(OH)_2_(CO_3_)_0.0125_·yH_2_O (Ni‐Mn/Al) layered double hydroxides (LDHs) are investigated to capture fluoride from aqueous media. The 2D mesoporous plate‐like Ni‐Mn/Al LDHs are successfully prepared via a co‐precipitation method. The molar ratio of divalent to trivalent cations is maintained at 3:1 and the pH at 10. The X‐ray diffraction (XRD) results confirm that the samples consist of pure LDH phases with a basal spacing of 7.66 to 7.72 Å, corresponding to the (003) planes at 2*θ* of 11.47^o^ and the average crystallite sizes of 4.13 to 8.67 nm. The plate‐like Mn‐doped Ni‐Al LDH consists of many superimposed nanosheets with a size of 9.99 nm. Energy‐dispersive X‐ray and X‐ray photoelectron spectroscopies confirm the incorporation of Mn^2+^ into the Ni‐Al LDH. UV–vis diffuse reflectance spectroscopy results indicate that incorporating Mn^2+^ into LDH enhances its interaction with light. The experimental data from the batch fluoride adsorption studies are subjected to kinetic models such as pseudo‐first order and pseudo‐second order. The kinetics of fluoride retention on Ni‐Mn/Al LDH obey the pseudo‐second‐order model. The Temkin equation well describes the equilibrium adsorption of fluoride. The results from the thermodynamic studies also indicate that fluoride adsorption is exothermic and spontaneous.

## Graphical abstract

Here, the structural, optical, and adsorptive behaviors of Ni_0.075_‐_x_Mn_x_Al_0.025_(OH)_2_(CO_3_)_0.0125_·yH2O (Ni‐Mn/Al) layered double hydroxides (LDHs) are investigated in the context of capturing fluoride from aqueous media.

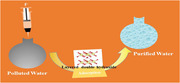

## 1 Introduction

Humans need water for drinking and activities like domestic, agricultural, industrial, etc. Currently, water bodies are being polluted by natural phenomena and daily human activities.^[^ ^1^ ^]^

Fluoride is one of the common contaminants of water, especially drinking water. Fluoride has a beneficial effect on teeth at concentrations below the WHO recommendation in drinking water, but excessive fluoride intake can lead to dental and bone disease. Exposure to fluoride above the guideline value of 1.5 mg L^−1^ recommended by WHO can lead to dental fluorosis.^[^ ^2^ ^]^ Groundwater fluoride levels are very severe in Rift Valley countries in East Africa, such as Tanzania, Kenya, and Ethiopia. High 2800 mg L^−1^ fluoride concentrations were reported in Lake Nakuru, Kenya. As in other African countries in the Rift Valley, fluoride is a significant health problem for communities using groundwater sources in the Rift valley region of Ethiopia.^[^ ^3^ ^]^ Therefore, it is essential to minimize or remove fluoride from the water.

Several physical, chemical, and biological methods, involving adsorption, biosorption, coagulation/flocculation, membrane filtration, and electrochemical methods have been commonly used to remove fluoride from aqueous solution. Among the mentioned strategies, adsorption is the method of choice because of its simple operation and design.^[^ ^4^, ^5^ ^]^ Here, the adsorption method using layered double hydroxides nanomaterials is employed for efficient performance.

The layered double hydroxides (LDHs) are an excellent multipurpose category of 2D inorganic layered materials having the general formula of${\left[M_{1-x}^{II}M_{x}^{III}{\left(OH\right)}_{2}\right]}^{x+}{\left(A^{n-}\right)}_{x/n}.yH_{2}O$, where M^II^, M^III^, A^n‐^ and x representing divalent cation, trivalent cation, anion and M^III^/(M^II^+M^III^) ratio, respectively. They have two main compositional parts the layer${\left[M_{1-x}^{II}M_{x}^{III}{\left(OH\right)}_{2}\right]}^{x+}$ and the interlayer(*A* ^*n* −^ )_*x*/*n*_.*yH*_2_ *O*. This gives LDHs with a generic layer system [LcDXLcD]_n_ in which L and D denote the layers of hydroxide anions, c represents the layers of metal cations, and X represents interlayers such as anions like CO_3_^2−^, Cl^−^, NO_3_^−^, SO_4_^2−^, etc. and neutral molecules like H_2_O. LDHs are also commonly known as anionic clay minerals due to interlayer anions. They can be found natural or synthetic.^[^ ^6^, ^7^, ^8^ ^]^

Promising results on the adsorption of fluoride from aqueous solution by these emerging 2D LDHs have been reported so far.^[^ ^9^, ^10^, ^11^ ^]^ Among these, the improved adsorption capacity of 84 mg F g^−1^ was reported by M. Dessalegne et al. 2016.^[^ ^11^ ^]^ The focus of most previous studies was on binary LDHs.^[^ ^1^, ^12^ ^]^ Ternary LDHs with even higher adsorption power has rarely been studied.^[^ ^13^, ^14^, ^15^, ^16^ ^]^ LDHs containing metals such as Mn, Al, etc., have a strong affinity for fluoride.^[^ ^13^ ^]^ While these previous studies have applied to defluoridation, few have used Mn‐containing LDHs.^[^ ^13^, ^15^ ^]^ They did not use Mn‐containing Ni‐Al LDHs to remove fluoride from the aqueous solution to the best of our knowledge.

Therefore, in this study, Mn‐doped Ni‐Al LDHs as Ni_0.075‐x_Mn_x_Al_0.025_(OH)_2_(CO_3_)_0.0125_·yH_2_O (x = 0, 0.00375, 0.0075, and 0.015) were synthesized from their respective inorganic salts by the coprecipitation method to remove fluoride from the aqueous solution. The structural features, compositions, and some properties of the synthesized LDHs have been investigated.

## 2 Results and Discussions

### 2.1 Crystal Structure and Chemical Compositions

**Figure 1:**
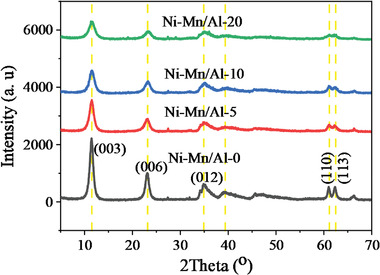
Powder XRD of Ni_0.075_Al_0.025_(OH)_2_(CO_3_)_0.0125_·yH_2_O (Ni‐Mn/Al‐0), Ni_0.07125_Mn_0.00375_Al_0.025_(OH)_2_(CO_3_)_0.0125_·yH_2_O (Ni‐Mn/Al‐5), Ni_0.0675_Mn_0.0075_Al_0.025_(OH)_2_(CO_3_)_0.0125_·yH_2_O (Ni‐Mn/Al‐10) and Ni_0.06_Mn_0.015_Al_0.025_(OH)_2_(CO_3_)_0.0125_·yH_2_O (Ni‐Mn/Al‐20) LDH samples.

The XRD patterns of the synthesized Ni‐Mn/Al LDHs are indicated in **Figure** **1**. Their well‐defined diffraction peaks at 11.47°, 23.1°, 34.86°, 39.3^o^, 61.0^o^, and 62.34° can be correlated to the Miller indices of (003), (006), (012), (015), (110) and (113) crystal planes (JCPDS: 00‐015‐0087).^[^ ^17^, ^18^ ^]^ These results of the XRD indicated the successful synthesis of Ni‐Mn/Al LDH, which is a hydrotalcite‐like structure known for intensive and sharper diffraction peaks at low 2*θ* in the range 11–23^o^, and broad asymmetric diffraction peaks at higher 2*θ* in the range of 34–66^o^. The appearance of the planes (012), (015), (018), and (110) in the sample represented the hexagonal structural lattice with rhombohedral 3R symmetry.^[^ ^19^ ^]^ As seen from diffraction patterns in Figure 1, as the amount of Mn dopant increased from 0% to 20%, the intensity of the peaks decreased, and some other low‐intensity peaks appeared at 2*θ* between 23 to 35 and peak at 2*θ* between 35 to 61 disappeared. It is well known that the radius of the Mn^2+^ cation ion is greater than the radius of the Ni^2+^ cation ion.^[^ ^20^ ^]^ This may prevent Mn from being incorporated into the host layer as its concentration increases resulting in the formation of other peaks.^[^ ^21^ ^]^

The *a* and *c* lattice parameters of the hexagonal structure of the synthesized LDH samples can be expressed as in the equation below:^[^ ^22^ ^]^

(1) $$\begin{array}{c} \frac{1}{d^{2}}=\frac{4}{3}\left(\frac{h^{2}+hk+k^{2}}{a^{2}}\right)+\frac{l^{2}}{c^{2}} \end{array}$$

where *d* represents the lattice spacing between two planes; *h*, *k*, and *l* represent the Miller indices of the planes. The cell parameters *c* and *a* of the hexagonal structure of the synthesized LDHs were calculated from the d‐spacing of the (003), (006), and (110) planes, respectively. The *c* parameter represented the thickness of three layers plus the interlayer space between them and is usually three times the *d*_003_ parameter. Herein the *c* parameter was obtained from two planes using the equation *c* = 3/2 [*d*_003_+2*d*_006_]. The lattice parameter, *a*, indicated an average distance between cations in the layer and was calculated from the (110) plane using *a = 2d*_(110)_, and the results are presented in **Table** **1**.^[^ ^12^, ^22^ ^]^ In general, the cell parameters decreased as the amount of manganese increased.

**Table 1:** The crystallographic parameters of the Ni‐Mn/Al LDH samples

LDH Samples	Basal spacing, [d_003_] Å	Lattice parameters [Å]		Average crystallite size, [nm]
		a	*c*	
Ni‐Mn/Al‐0	7.72	3.04	23.13	8.67
Ni‐Mn/Al‐5	7.71	3.0	23.15	5.16
Ni‐Mn/Al‐10	7.66	3.04	22.98	6.27
Ni‐Mn/Al‐20	7.67	3.0	22.97	4.13

The average crystallite size of the synthesized LDHs was calculated from the full width at half‐maximum (FWHM) of the diffraction peaks at the (003), (006), (012), and (110) planes. The Debye‐Scherrer equation was used to approximate the crystallite size of the synthesized LDHs from the significant diffraction peaks utilizing the equation:^[^ ^23^, ^24^ ^]^

(2) $$\begin{array}{c} D=\frac{K\lambda }{\beta \cos\theta } \end{array}$$

where the average crystallite size, the wavelength of the X‐ray radiation (Cu K*α* = 0.15418 nm), the Scherrer constant 0.9, the FWHM, and the Bragg's diffraction angle are represented by D, *λ*, K, *β* and *θ*, respectively. The average crystallite sizes of the Ni‐Mn/Al LDHs were between 4.13 and 8.67 nm. The *a* and *c* parameters of the Ni‐Mn/Al LDHs obtained in this study were in agreement with the reported literature.^[^ ^12^, ^18^ ^]^

**Figure 2:**
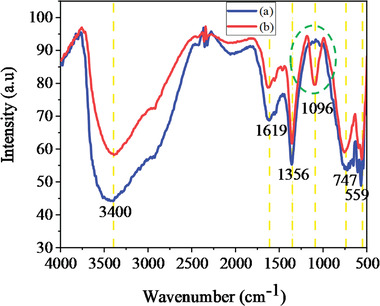
FT‐IR spectrum of the synthesized a) Ni‐Mn/Al‐0 and b) Ni‐Mn/Al‐10 LDHs.

The FT‐IR was also used to investigate the chemical compositions of the Ni‐Mn/Al LDH samples, and their spectra are shown in **Figure** **2**. The prominent FT‐IR absorption band at 3400 cm^−1^ can be assigned to the stretching vibrations of the hydroxyl groups of the metal hydroxide layers, interlayer water molecules, and hydrogen bonding among hydroxyl groups.^[^ ^25^, ^26^ ^]^ The absorption band at ≈1620 cm^−1^ can be due to the bending mode of the H_2_O molecules.^[^ ^26^, ^27^ ^]^ The two absorption bands at 1356 cm^−1^ can be ascribed to the vibration of the interlayer carbonate anion.^[^ ^19^, ^26^, ^27^, ^28^ ^]^ The extraordinary peak at ≈1096 cm^−1^ in the case of Ni‐Mn/Al‐10 LDH can be specifically attributed to the O‐H bending vibration coordinated to Mn.^[^ ^29^, ^30^ ^]^ The peaks within the range of 500–900 cm^−1^ in the low‐frequency region can generally be regarded as metal‐O‐metal and O‐metal‐O bending vibration peaks like the vibrational manner of Al–O or Ni–O or Mn–O.^[^ ^19^, ^27^, ^31^ ^]^ Therefore, the FTIR result verifies the advent of a new chemical feature associated with the incorporation of Mn.

**Figure 3:**
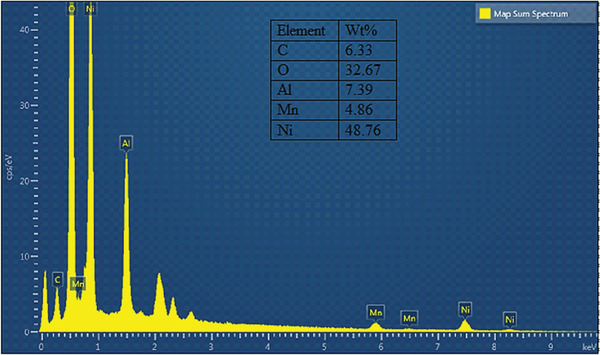
EDS images of the synthesized Ni‐Mn/Al‐10 LDH.

The elemental composition of the Ni‐Mn/Al‐10 LDH was investigated by energy dispersive X‐ray spectrometry (EDS). As shown in **Figure** **3**, the surface layer of this synthesized Mn‐doped Ni‐Al LDH was composed of Ni, Mn, Al, C, and O elements. According to the relative compositions of Ni, Mn, and Al elements from the EDS result, the molar ratio of divalent (Ni^2+^ + Mn^2+^) to trivalent (Al^3+^) was obtained to be 3.4:1 and is close to the initially taken stoichiometric ratio of 3:1 of divalent to trivalent in Ni‐Mn/Al‐10 LDH during the synthesis. The molar ratio of Mn^2+^ to (Ni^2+^ + Mn^2+^) was found to be 0.097 and is also close to the initially taken proportion of Mn^2+^/(Ni^2+^ + Mn^2+^) = 0.1 during the synthesis. So, the EDS analysis (together with XRD results) demonstrated that the Ni‐Mn/Al LDHs were synthesized successfully by the coprecipitation method.

**Figure 4:**
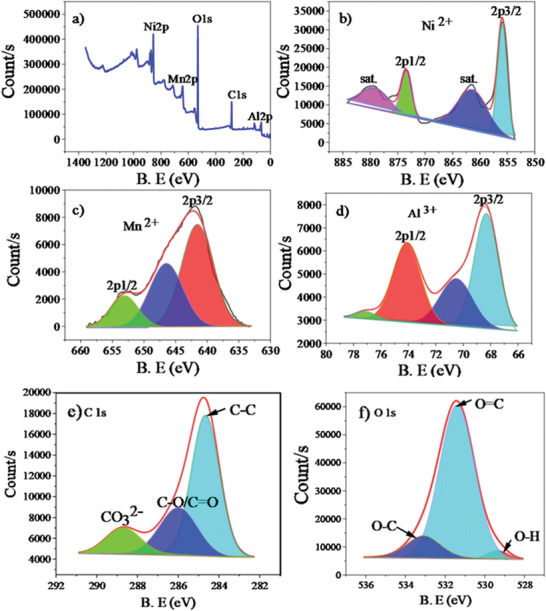
The XPS spectra of Ni‐Mn/Al‐10 LDH: XPS spectrum of survey scan a) and High‐resolution XPS spectrum of Ni 2p b), Mn 2p c), Al 2p d), C 1s e), and O 1s f).

The XPS was used to evaluate the chemical compositions and the oxidation states of the elements on the surface of the synthesized Ni‐Mn/Al‐10 LDH, as indicated in **Figure** **4**. It also assures the bonding of containing atoms in the LDHs. The survey scan spectrum of the LDH in Figure 4a showed the presence of Ni, Mn, Al, O, and C elements. The Ni 2p high‐resolution XPS spectrum indicated in Figure 4b was fitted to two prominent peaks, one spin‐orbit doublet, and two satellite peaks. The spin‐orbital doublet peaks at the binding energy of 855.86 and 873.47 eV with a spin‐energy separation of 17.6 eV can be designated as the divalent form of nickel (Ni^2+^). The two satellite peaks were found at 861.38 and 879.18 eV binding energy. The result agrees well with the previously reported literature.^[^ ^28^, ^32^ ^]^ The high‐resolution Mn 2p XPS spectrum of Mn‐doped LDH in Figure 4c is conquered with two peaks at a binding energy of 641.58 and 653.37 eV attributed to spin‐orbit characteristics of Mn^2+^, accounting for Mn has replaced Ni in the LDH layer region.^[^ ^32^, ^33^ ^]^ The Al 2p high‐resolution XPS spectrum in Figure 4d provided the two peaks at 68.42 and 74.08 eV, which can be assigned to Al 2p3/2 and Al 2p1/2, respectively.^[^ ^28^ ^]^ The C 1s high‐resolution XPS spectrum in Figure 4e was fitted to three peaks at 284.76, 286.39, and 288.54 eV. The central peak at 284.76 eV corresponds to C—C coordination. The peak at 286.39 eV is a characteristic of C—O, and C=O. The peak at 288.54 eV can be attributed to carbonate species in the interlayer.^[^ ^19^, ^28^, ^34^ ^]^ The O 1s high‐resolution XPS spectrum in Mn‐doped LDH indicated in Figure 4f assumed the existence of O—H, O=C, and O—C at 529.34, 531.41 and 533.18 eV, respectively.^[^ ^19^, ^34^ ^]^ Therefore, it can be said that Mn was doped in Ni‐Al LDH and exists in an oxidation state of +2. The presence of Mn can aid the defluorination process. This XPS analysis was consistent with the EDS spectrum in corroborating the compositions of the Ni‐Mn/Al LDH samples.

### 2.2 Morphology

**Figure 5:**
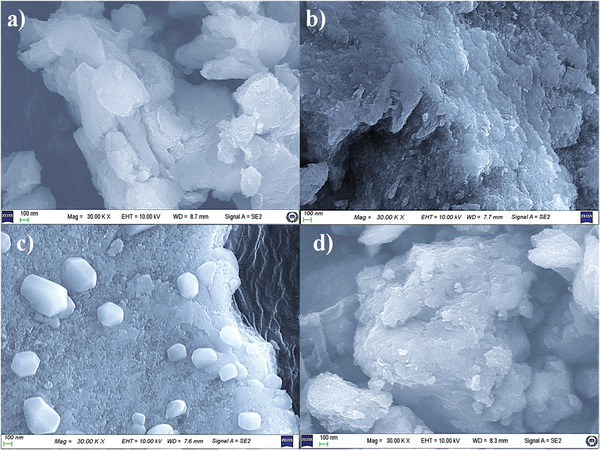
SEM micrographs of the co‐precipitated a) Ni‐Mn/Al‐0 b) Ni‐Mn/Al‐5 c) Ni‐Mn/Al‐10, and d) Ni‐Mn/Al‐20 LDHs.

The surface morphology of the synthesized Ni‐Mn/Al LDH samples was investigated by SEM from the sample surface at different magnifications. **Figure** **5** indicates the SEM micrographs of the synthesized Ni‐Mn/Al LDHs. The micrographs suggest that the synthesized LDHs were found to be aggregates of the plate‐like structures grown overlapping to form hexagonal blocks with varying sizes. These obtained structures were similar to the previously reported LDHs.^[^ ^35^, ^36^ ^]^

**Figure 6:**
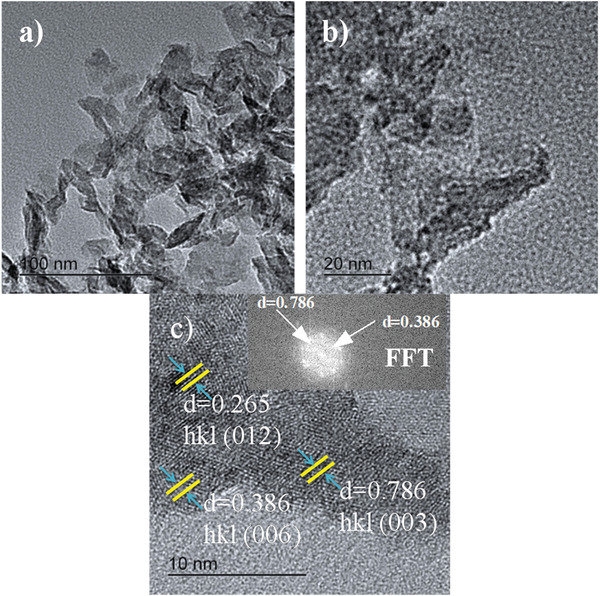
TEM a) and HRTEM b,c) images of the synthesized Ni‐Mn/Al‐10 LDH.

The TEM and HRTEM images of the Ni_0.0675_Mn_0.0075_Al_0.025_(OH)_2_(CO_3_)_0.0125_·yH_2_O LDH samples are shown in **Figure** **6**. By investigating different regions of the TEM images of the Mn‐doped LDH sample, nano‐plates with well‐defined morphology of stacked multilayer structure were observed (Figure 6a). From the HRTEM, it was also observed that the individual nano‐plates consist of several nano‐particles (Figure 6b). The hexagonal plate‐like Mn‐doped LDH consisted of many superimposed nanosheets with the size of ≈9.99 nm. Furthermore, the HRTEM results showed lattice fringes with the d‐spacing of 0.786, 0.386, and 0.265, corresponding to the (003), (006), and (012) planes, respectively (Figure 6c), confirming plate‐like particles with the hexagonal structural phase that indicates the layered structure of Ni_0.075‐x_Mn_x_Al_0.025_(OH)_2_(CO_3_)_0.0125_·yH_2_O LDH is similar to the investigation by XRD (Figure 1) and SEM (Figure 5).^[^ ^37^, ^38^ ^]^

### 2.3 Optical Properties

**Figure 7:**
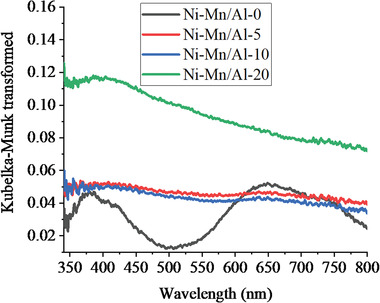
Kubelka‐Munk transformed UV–vis diffuse reflectance spectra of Ni‐Mn/Al LDHs.

The optical absorption spectra of Ni‐Mn/Al LDHs were studied by UV–vis‐DRS, and the ranges are shown in **Figure** **7**. As shown in Figure 7, in the case of Ni‐Mn/Al‐0 LDH, there are two prominent absorption peaks located ≈350 to 400 nm and 650 to 700 nm, ascribed to the d‐d transition of Ni^2+^ in the octahedral geometry and the existence of CO_3_^2−^ ions in LDH interlayer. The spectra of Ni‐Mn/Al‐5, Ni‐Mn/Al‐10, and Ni‐Mn/Al‐20 LDHs were nearly straight lines in the range of 200 to 800 nm, which indicates full‐spectrum absorption probably due to their characteristic dark‐gray color.^[^ ^39^ ^]^ The spectrum of Ni‐Mn/Al‐20 LDH was slightly curved in the range from 350 to 450 nm, which might be because of a few agglomerations of MnO outside LDH as the amount of Mn increased. It can be found that the response to light of Ni‐Mn/Al LDHs is sensitive in both ultraviolet and visible light regions, which is the aggregate effect between Mn^2+^ and Ni_0.075_Al_0.025_(OH)_2_(CO_3_)_0.0125_·yH_2_O LDH.

### 2.4 Thermal Property by TG‐DCS

**Figure 8:**
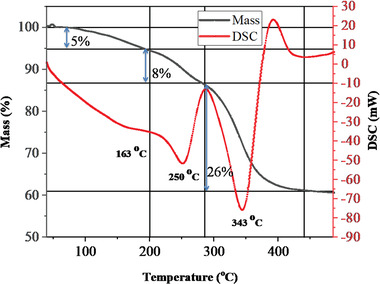
TG‐DSC curve of Ni‐Mn/Al‐10 LDH.

The TG‐DCS curve of the prepared Ni‐Mn/Al‐10 LDH sample is displayed in **Figure** **8**. As seen from this thermogram, the decomposition of the Mn‐doped LDH sample resulted in three‐step weight loss peaks (two prominent peaks and one shoulder peak). The endothermic peaks were observed correspondingly on the DSC curve. A total of ≈39% weight loss occurred. The initial mass loss of ≈5% happened at the endothermic peak of 163 °C (30‒200 °C). This weight loss may be due to the elimination of adsorbed and interlayer water, which was negligible due to the thorough drying of the sample. The second stage mass loss of ≈8% happened at ≈250 °C endothermic peak (200‒285 °C), and this could be due to dehydroxylation of the brucite‐like metal hydroxide layers. The final stage of mass loss of the sample occurred at ≈343 °C endothermic peaks (290‒440 °C) and resulted in a mass loss of ≈26%. Most of the mass loss of the sample has occurred at this stage and can be due to the decomposition of interlayer CO_3_^2−^ anions. Above 450 °C, the brucite‐type structure of the synthesized Mn‐doped LDH sample collapsed, resulting in mixed metal oxides. No marked weight change after 450 °C has happened, demonstrating that the conversion from LDH to oxides was finished.^[^ ^26^, ^40^, ^41^ ^]^

### 2.5 Specific Surface Area and Porosity Measurement by BET

Adsorbent properties such as specific surface area and pore sizes influence its adsorption. The larger specific surface area of the adsorbent provides more active sites and sufficient attachment sites, whereas the pore size determines the diffusion rate of the adsorbate into the adsorbent.^[^ ^39^ ^]^ Hence, the porous structure, surface area, pore volume, and pore size distribution of the 2D mesoporous plate‐like Ni‐Mn/Al‐10 LDH was tested using the BET method in the N_2_ adsorption‐desorption environment (**Table** **2**). As‐synthesized 2D mesoporous plate‐like Mn‐doped LDH exhibited a narrow pore size of 9.24 nm, a pore volume of 0.033 cc g^−1^, and a specific surface area of 45.11 m^2^ g^−1^ (Table 2). The pore size distribution of the Mn‐doped LDH lay between 2–20 nm, indicating the existence of mesopores.^[^ ^37^, ^42^ ^]^

**Table 2:** BET textural characteristics

Samples	Surface area [m^2^ g^−1^]	Pore volume [cm^3^ g^−1^]	Average pore size [nm]
Ni‐Mn/Al‐00	2.30	0.0015	13.44
Ni‐Mn/Al‐05	45.11	0.033	9.24

### 2.6 Aqueous Fluoride Adsorption

#### 2.6.1 The Effect of the Dopant Amount

**Figure 9:**
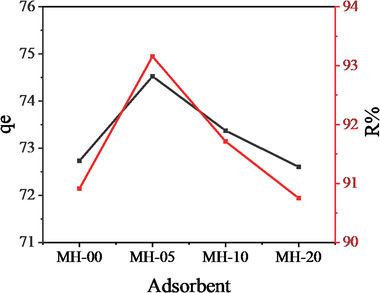
Aqueous fluoride adsorption performances of Ni‐Mn/Al LDHs.

Here the effect of Mn^2+^ dopant on the adsorption performance of fluoride from aqueous solution onto Ni‐Mn/Al LDHs under the same conditions at 25 °C, 45 min, pH 7, and 150 rpm was studied. The results are displayed in **Figure** **9**. As indicated in the figure, the adsorption capacity (q_e_) of Ni‐Mn/Al‐0, Ni‐Mn/Al‐5, Ni‐Mn/Al‐10, and Ni‐Mn/Al‐20 was found to be 72.73, 74.74, 73.37 and 72.60 mg g^−1^, respectively. Although the differences are slight, the amount of fluoride adsorption on Ni‐Mn/Al‐5 was higher than the others. Similarly, a high percentage of fluoride removal/ removal efficiency (%R) was obtained by this Mn‐doped Ni‐Al LDH. This may be due to the hydrotalcite properties of the material in which the layered structure is more pronounced at this dopant concentration, as shown in the XRD results in addition to the dopant effect. The decrease in the removal efficiency as the dopant amount increased from 5% to 20% might be due to the aggregation of the adsorbent leading to the rigidity of the LDHs, as can be seen from the SEM results. The rigid structure prevents accessibility to the interlayers and the external surface of LDHs.^[^ ^22^ ^]^ Therefore, Ni‐Mn/Al‐5 LDH was used as an adsorbent to study the influence of different parameters such as pH, initial concentration, adsorbent amount, time, and temperature on its adsorption performance.

#### 2.6.2 Adsorption Kinetics and Influence of Contact Time on the Adsorption Capacity

**Figure 10:**
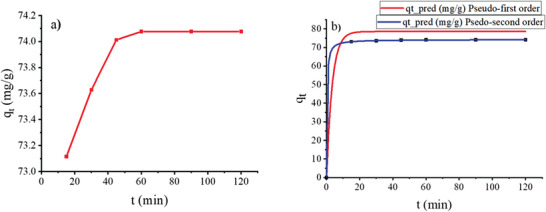
a) Influence of contact time on fluoride adsorption onto Ni‐Mn/Al‐5 LDH and b) pseudo‐first and pseudo‐second order kinetic modeling.

The influence of contact time on the amount of fluoride adsorbed on the Ni‐Mn/Al‐5 LDH was studied. **Figure** **10** shows fluoride uptake, q_t_ (mg g^−1^), versus time (min) for fluoride adsorption kinetics at 25 °C. The fluoride removal of Mn‐doped LDH was initially rapid, and no significant increase was observed after 45 min.

To characterize the sorption behavior of fluoride in an aqueous solution over time, two kinetic models, namely pseudo‐first order and pseudo‐second order, were investigated. Results of fluoride removal kinetics onto Mn‐doped LDH are shown in Figure 10. Kinetic modeling was performed using nonlinear regression according to pseudo‐first and pseudo‐second order equations in CAVS software that gave the results listed in **Table** **3**. The pseudo‐second‐order kinetic model indicated that the equilibrium sorption capacity, q_e_, obtained from the kinetic equation (calculated adsorption capacity) (74.3 mg g^−1^) was close to that obtained from the equilibrium study (experimental adsorption capacity) (74.077 mg g^−1^) for the initial fluoride concentration of 20 mg L^−1^. Thus, it can be stated that the pseudo‐second‐order kinetic model fitted the fluoride adsorption process well.

**Table 3:** Outputs of the kinetic models fitted for aqueous fluoride adsorption data at a temperature of 25 °C and adsorbent amount of 0.01 g/40 mL

Kinetics models	Parameters	Ni‐Mn/Al‐5
PFO	q_e_	73.978
	K_1_	0.296
	R^2^	0.9999
PSO	q_e_	74.304
	K_2_	0.055
	R^2^	0.99999

Since fluoride adsorption followed pseudo‐second‐order kinetics, this may indicate that boundary layer resistance was not the rate‐limiting step. Therefore, the rate of fluoride adsorption can be primarily controlled by the chemisorption process associated with the chemical characteristics of LDH and fluoride molecules that involves the sharing of electrons between them, usually confined to only one layer of molecules on the surface.^[^ ^43^ ^]^

#### 2.6.3 Adsorption Isotherm and Influence of Initial Concentration on the Adsorption Capacity

**Figure 11:**
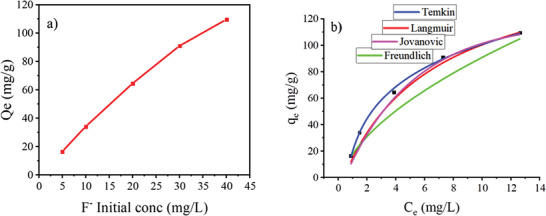
a) The influence of initial fluoride concentration and b) Isotherm models fitted to the adsorption of fluoride onto Ni‐Mn/Al‐5 LDH.

The equilibrium results obtained at 25 °C, 45 min, 150 rpm, and ≈pH 7 for five different aqueous fluoride concentrations to determine the effect of initial fluoride concentration are given in **Figure** **11**. As indicated in Figure 11, when the initial fluoride concentration increased, the adsorption capacity of Ni‐Mn/Al‐5 LDH gradually increased from 16.48 to 109.53 mg g^−1^. The adsorption capacity increases with increasing the initial fluoride concentration as the driving force is likely to increase in solution, which induces mass transfer effects.^[^ ^44^ ^]^

The parameters of used isotherm equations are listed in **Table** **4**. Isotherm models such as Langmuir, Freundlich, Temkin, and Jovanovic were applied to identify the influence of initial fluoride concentration on the sorption process. The equilibrium adsorption capacities were calculated according to Equation (3) based on the experimental data. The maximum adsorption capacity, q_max_, listed in Table 4 exceeds that obtained experimentally, indicating that the studied adsorbent can retain more significant quantities of fluoride than those in the solution. In terms of the values of the correlation coefficients, R^2^, listed in Table 4, which are goodness‐of‐fit measures and confirm the representativeness of the experimental data, the Temkin model provided a better fit to the experimental results than the other models tested (Figure 11b).

**Table 4:** Outputs of the equilibrium isotherm models fitted for aqueous fluoride equilibrium adsorption data at a temperature of 25 °C and adsorbent amount of 0.01 g/40 mL

Isotherm models	Parameters	Ni‐Mn/Al‐5
Langmuir	q_max_	162.84
	K_L_	0.168
	R^2^	0.991
Freundlich	K_f_	27.784
	N	1.785
	R^2^	0.937
Temkin	K_T_	1.776
	B_T_	70.75
	R^2^	0.996
Jovanovic	q_m_	117.94
	K_j_	0.206
	R^2^	0.993

The regression (R^2^) of the Temkin isotherm plot of fluoride adsorption onto Ni‐Mn/Al‐5 LDH was found to be 0.996 (Table 4), indicating the interactions between adsorbate and adsorbent likely play a vital role in the fluoride adsorption process. Notably, the equilibrium binding constant (k_T_) was also significant. This indicated that this LDH could form a strong bond with fluoride.^[^ ^45^ ^]^ The positivity of the B_T_ value means that the adsorption processes were exothermic and predominantly physical.^[^ ^4^ ^]^

#### 2.6.4 Influence of Adsorbent Amount on the Adsorption Efficiency

**Figure 12:**
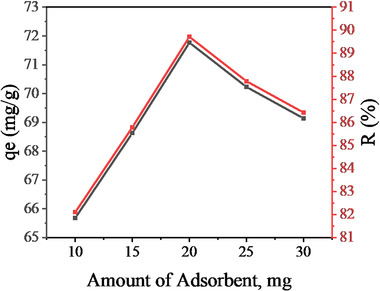
Effect of Ni‐Mn/Al‐5 LDH adsorbent on the aqueous fluoride removal efficiency and adsorption capacity.

The influence of adsorbent mass on the adsorption capacity and removal efficiency of fluoride from aqueous solution onto Ni‐Mn/Al‐5 was studied, and the results are shown in **Figure** **12**. The effect of an adsorbent amount in the range of 10–30 mg at a stirring speed of 150 rpm, pH 7, contact time of 45 min, and reaction temperature of 25 °C was investigated using 40 mL of fluoride at an initial concentration of 20 mg L^−1^. Figure 12 shows that the removal percentage of fluoride increased significantly from 82.1% to 89.95%, and the adsorption capacity increased from 65.68 to 71.77 mg g^−1^ when the adsorbent amount increased from 10 to 20 mg, then slightly decreased as the adsorbent amount reached 30 mg. This is because the energy sites on the adsorbent vary according to the amount of adsorbent, and the sorption efficiency changes accordingly. Therefore, as the amount of adsorbent increases, more surface area is made available for the sorption process, and the sorption efficiency increases to a certain limit. An excessive increase in the amount of adsorbent creates a resistance to the mass transfer of fluoride ions between the aqueous and solid phases, and consequently reduces the number of adsorption sites available for adsorption. The increase in the adsorbent dose might also cause aggregation of adsorbent, and consequently, the available adsorption sites might decrease.^[^ ^13^ ^]^

#### 2.6.5 Influence of pH on the Adsorption Efficiency

**Figure 13:**
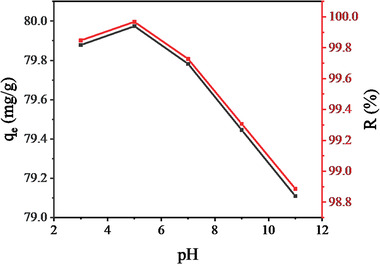
The influence of pH on the removal efficiency and adsorption capacity of aqueous fluoride onto Ni‐Mn/Al‐5 LDH.

The pH of the solution is one of the critical parameters controlling the adsorption efficiency. The adsorption experiment of aqueous fluoride by Ni‐Mn/Al‐5 LDH was carried out with the pH values from 3 to 11 at the initial fluoride concentration of 20 mg L^−1^, adsorbent dose of 0.01 g/40 mL, 25 °C, and 45 min. The influence of initial pH values on fluoride adsorption in an aqueous solution is given in **Figure** **13**. The adsorption efficiency reached their maximum at pH 5, and beyond this, it gradually decreased.

The adsorption efficiency can be explained by the charge properties of the LDH surface as it interacts with the aqueous solution. With the positive charge resulting from the isomorphic substitution, LDH acquires other expenses due to the adsorption of ions from aqueous, like H^+^ or O^•H•−^, which is greatly influenced by the solution pH. In this case, at pH > 5, the surface of LDH is negatively charged owed to the presence of more O^.H.−^ in the solution, which could compete with fluoride, whereas at pH < 5, the surface of LDH becomes positively charged as a result of more H^+^ (proton) in the solution. Therefore, this LDH prefers the adsorption of fluoride molecules at a pH below 5. Indeed, with a pH value of ≈5, good fluoride adsorption was observed on the LDH material. This was because at low pH, the hydroxyl groups on the surface of LDHs were protonated, and the electrostatic interaction dominated fluoride adsorption.^[^ ^9^ ^]^ However, the removal efficiency decreased with increasing pH, primarily due to electrostatic repulsion between similarly charged species.^[^ ^46^ ^]^

#### 2.6.6 Thermodynamics and Temperature Influence the Adsorption Efficiency

**Figure 14:**
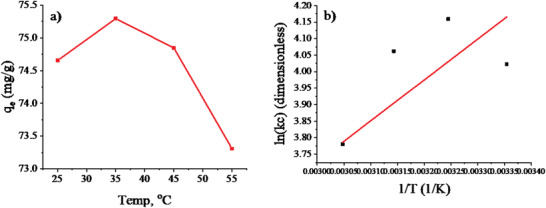
Dependence of q_e_ on temperature and its van't Hoff plot.

Whether the process is exothermic or endothermic, the influence of temperature plays an essential role in the sorption process. To investigate the effect of temperature on fluoride adsorption efficiency (20 mg L^−1^) on Mn‐doped LDH (0.01 g/40 mL); experiments were conducted in the range of 25 to 55 °C. The temperature dependence of q_e_ is shown in **Figure** **14**. As the temperature of the solution increased to 35 °C, the q_e_ of LDH increased somewhat, and beyond this, it decreased strongly.

**Table** **5** presents the thermodynamic parameters obtained from the thermodynamic data of the sorbent material, Ni‐Mn/Al‐5 LDH, at temperatures of 25, 35, 45, and 55 °C using van't Hoff plot in CAVS software. The negative value of ΔG^o^ indicates that the sorption of fluoride onto the sorbent was spontaneous under the experimental conditions and thermodynamically feasible. The ΔG° for physisorption ranges from 0 to −20 kJ mol^−1^, while chemisorption occurs in the range −80 to −400 kJ mol^−1^. The calculated ΔG^o^ values indicate the interaction between fluoride and Ni‐Mn/Al‐5 LDH can be considered a physical process.^[^ ^4^, ^47^ ^]^

**Table 5:** Thermodynamic Parameters of the Adsorption of Fluoride onto Ni‐Mn/Al‐5 LDH

Kc	T [°C]	Delta G [KJ mol^−1^]	Delta H [KJ mol^−1^]	Delta S [J mol^−1^]
55.86	25	−9.97	−6.49	12.55
64.011	35	−10.66		
58.09	45	−10.74		
43.82	55	−10.31		

The negative value of ΔH^o^ of fluoride adsorption confirms that the process is exothermic and proceeds well at low temperatures. So, the amount adsorbed at equilibrium decreased with increasing temperature. The positive value of ΔS^o^ more likely confirms the selectivity of fluoride on the surface of Ni‐Mn/Al‐5 LDH. It also suggests the possibility of structural changes or rearrangements in the fluoride–LDH interaction due to increased random effects of their interface.^[^ ^4^, ^44^ ^]^

## 3 Conclusion

The Ni_0.075‐x_Mn_x_Al_0.025_(OH)_2_(CO_3_)_0.0125_·yH_2_O LDH has been produced successfully using the co‐precipitation method at low temperature being the pH kept at 10 for efficient aqueous fluoride adsorption. The XRD, TEM, HR‐TEM, XPS, EDS, and FTIR studies confirmed the formation of the brucite‐like structure of the synthesized Ni_0.075‐x_Mn_x_Al_0.025_(OH)_2_(CO_3_)_0.0125_·yH_2_O LDHs. The XRD data of the Mn‐doped Ni‐Al LDH provided the value of interlayer/basal spacing to be 7.66 to 7.71 Å indicating a layered structure. The EDS and XPS studies confirmed that the Mn was doped in Ni‐Al LDH in the form of Mn^2+^ oxidation state. The HRTEM studies revealed that particles with plate‐like shapes were obtained. The UV–vis‐DRS results showed that doping Mn^2+^ into Ni‐Al LDH increased the interaction of LDH with light, where the absorption of visible light increased with increasing Mn concentration. According to the batch adsorption experiments of fluoride removal from aqueous solution by Mn‐doped Ni‐Al LDH, the kinetics of fluoride adsorption onto Mn‐doped Ni‐Al LDH was precisely described by pseudo‐second‐order model, while its adsorption isotherm was more reasonably fitted with Temkin model. From the Langmuir adsorption isotherm, the maximum adsorption capacity was found to be 162.84 mg g^−1^. The van't Hoff plot specified that the adsorption process was spontaneous and exothermic. It can be concluded that Mn‐doped Ni‐Al LDH has great application prospects in drinking water defluorination.

## 4 Experimental Section

### Materials

NiCl_2_.6H_2_O (97%, LOBA CHEMIE PVT. LTD), MnSO_4_.H_2_O (98%, SAMIR Tech‐chem PVT. LTD), and AlCl_3_.6H_2_O (98%, CDH(P) Ltd) were used as starting materials in the preparation of Ni‐Mn/Al LDHs. NaF for aqueous fluoride solution preparation and Eriochrome Black T (EBT) for complexing. NaOH and HCl were used to adjust the pH.

### Synthesis

In this study, the co‐precipitation method was used to synthesize a series of Ni‐Mn/Al LDHs with different molar ratios of Ni^2+^/Mn^2+^. According to the molar ratio of M^II^/M^III^ = 3.0, the first solution was prepared by dissolving the desired amount of NiCl_2_.6H_2_O, MnSO_4_.H_2_O, and AlCl_3_.6H_2_O in deionized water and labeled “Sol A.” The precipitating agent, a mixture of 2 m NaOH and 1 m NaHCO_3_, was also prepared and designated “Sol B.” “Sol B” was then added dropwise to “Sol A” while maintaining pH at 10 under vigorous stirring. The resulting slurry was aged at 80 °C for 3 h. Then the precipitate was filtered and washed until the pH dropped below eight and dried at 110 °C for 15 h. Changing the content of Mn in the LDH and the above method was repeated to prepare Ni‐Mn/Al LDHs with different Ni^2+^/Mn^2+^ molar ratios. Finally, the synthesized samples were powdered and stored at room temperature for further characterization and applications.^[^ ^22^, ^41^ ^]^

### Characterizations

The synthesized Ni‐Mn/Al LDH samples were recorded with Bragg‐Brentano geometry (*θ*‐2*θ* scans) on an X‐ray diffractometer (XRD‐7000, SHIMADZU Corporation, Japan) to obtain the XRD patterns. Cu K*α* (*λ* = 1.54 Å) was used as the beam source. The XRD peaks were recorded from 5 to 80^o^ 2*θ* in steps of 0.02^o^ 2*θ*, with a measuring time of 0.4 s per step. The crystallographic phase of the synthesized LDHs was investigated using QualX2 software with Cu/K*α* radiation (40 kV, 30 mA) at 0.02° step size.

The functional groups of the synthesized LDHs were studied by a VERTEX 70 DTGS infrared spectrometer (FT‐IR) in the range of 4000–400 cm^−1^ at a resolution of 4 cm^−1^ done by mixing LDH with KBr at a weight ratio of 1:100 after pellet formation.

A ZEISS Gemini SEM equipped with an energy‐dispersive X‐ray analyzer at an accelerating voltage of 10 kV was used to observe the morphology of the synthesized LDHs by SEM and EDS to determine the approximate composition of the samples.

The optical properties of the synthesized LDHs were studied by UV–vis diffuse reflectance spectroscopy (UV–vis DRS) of a UV‐3600Plus Series (SHIMADZU) using BaSO_4_ as reference.

Thermal analysis (TGA/DSC) of the LDHs was examined by a SETARAM LABSYS EVO instrument with a linear heating rate of 10 °C min^−1^ from room temperature up to 900 °C, and air atmosphere using an Al_2_O_3_ crucible, and a sample amount of ≈10 mg.

The surface area and porosity analysis of the synthesized Ni‐Mn/Al LDH sample was measured with an N_2_ adsorption‐desorption technique at 77 K based on the Brunauer–Emmett–Teller (BET) isotherm model (Quantacrome Instruments version 11.0).

### Aqueous Fluoride Adsorption Experiment

Aqueous fluoride adsorption was investigated by batch adsorption experiments in which 10 mg of synthesized Ni‐Mn/Al LDH was mixed with 40 mL of 20 mg L^−1^ initial concentration of aqueous fluoride solution and the suspension was shaken at a rate of 150 rpm. Experiments were done in duplicate, and mean values were recorded. The effects of adsorption factors such as pH, initial concentration, adsorbent amount, time, and adsorption temperature were studied, and the used values of these experimental conditions were presented in **Table** **6**. The equilibrium isotherms were studied by varying the fluoride concentration, the adsorption kinetics by varying the adsorption time and thermodynamics by changing the temperature.

**Table 6:** Experimental parameters and their values for aqueous fluoride adsorption

Adsorption Parameters	Values
Contact time (min.)	15, 30, 45^a)^, 60, 90, 120
Initial fluoride concentration (mg/L)	5, 10, 20^a)^, 30, 40
Temperature (°C)	25^a)^, 35, 45, 55
pH	3, 5, 7^a)^, 9, 11
Adsorbent amount (mg)	10^a)^, 15, 20, 25, 30

^a)^ Selected values when studying the effect of parameters on the adsorption process.

After adsorption, the amount of fluoride remaining in the solution (Ce) was evaluated by a UV–vis spectrophotometer based on the Al‐EBT complexing method at a wavelength of 542 nm after filtering out the adsorbent. The standard calibration curve was obtained by analyzing known concentrations of fluoride solutions at 0.5, 1, 2, and 3 ppm. A linear regression equation y = 0.0312x + 0.0163 with R^2^ = 0.999 was obtained, from which the unknown fluoride concentration was derived.

The adsorbed amount of fluoride was calculated from the following equation:^[^ ^48^ ^]^

(3) $$\begin{array}{c} q_{e}=\frac{\left(\mathrm{Co}-\mathrm{Ce}\right)V}{M} \end{array}$$

where q_e_ was fluoride adsorption capacity (mg g^−1^) at time t, Co and Ce were the initial and equilibrium fluoride concentrations, respectively, *V* was the volume (mL) of fluoride solution, and *M* was the mass (mg) of adsorbent.

The removal efficiency was calculated using the equation.^[^ ^48^ ^]^

(4) $$\begin{array}{c} R\;\left(\%\right)=\frac{\left(\mathrm{Co}-\mathrm{Ct}\right)}{\mathrm{Co}}\times 100 \end{array}$$

where Co (mg L^−1^) was the initial fluoride concentration and Ct (mg L^−1^) was the fluoride concentration at time t, respectively.

### Adsorption Kinetics, Isotherm, and Thermodynamic Modeling

Kinetics of fluoride adsorption in aqueous onto Ni‐Mn/Al LDH was analyzed with nonlinear models such as pseudo‐first‐order (PFO) model and pseudo‐second‐order (PSO) model, and their equations were given in **Table** **7**. Lagergren's kinetic equation (PFO model) based on solid capacity had been used extensively to describe liquid–reliable adsorption systems. The PSO model was also successfully applied to absorb pollutants in aqueous solutions, such as metal ions, dyes, herbicides, oils, and organic substances. Once the sorption kinetics reached equilibrium, the nonlinear isotherm models of Langmuir, Freundlich, Temkin, and Jovanovic were applied. Their equations were also given in Table 7. The Langmuir isotherm refers to monolayer adsorption by energetically identical sites, whereas the Freundlich isotherm describes heterogeneous surfaces and does not assume monolayer capacity.^[^ ^49^ ^]^ The Temkin isotherm was often applied to describe the adsorbate‐adsorbent interaction, which was the adsorption potential of the adsorbate on the adsorbent surface. A uniform distribution of binding energies defines it.^[^ ^50^, ^51^ ^]^ The Jovanovic model was based on the assumptions used in the Langmuir model, but in addition, there was the possibility of some mechanical interactions between adsorbates and adsorbents.^[^ ^5^ ^]^

**Table 7:** Kinetics, isotherm, and thermodynamic models and their equation^[^ ^5^, ^49^, ^52^ ^]^

Model types		Expressions
Kinetic models	PFO	$q_{t}=q_{e}-e^{k_{1}\times t}\;\;\;\;\;\;\;\;\;\;\;\;\;\;\;\;\;\;\;\;\;\;\;\;\;\;\;\;\;\;\;\;\;\left(5\right)$
	PSO	$q_{t}=\frac{k_{2}\times q_{e}^{2}\times t}{1+k_{2}\times q_{e}\times t}\;\;\;\;\;\;\;\;\;\;\;\;\;\;\;\;\;\;\;\;\;\;\;\;\;\left(6\right)$
Isotherm models	Langmuir	$q_{e}=\frac{K_{L}C_{e}}{1+b_{L}C_{e}}\;\;\;\;\;\;\;\;\;\;\;\;\;\;\;\;\;\;\;\;\;\;\;\;\;\;\;\;\left(7\right)$
	Freundlich	$q_{e}=K_{F}C_{e}^{a_{F}}\;\;\;\;\;\;\;\;\;\;\;\;\;\;\;\;\;\;\;\;\;\;\;\;\;\;\;\;\;\;\;\left(8\right)$
	Temkin	$q_{e}=\frac{RT}{b_{T}}\left(\ln A_{T}C_{e}\right)\;\;\;\;\;\;\;\;\;\;\;\;\;\;\;\;\;\;\;\;\;\;\;\;\;\left(9\right)$
	Jovanovic	$q_{e}=q_{m}\left(1-e^{-K_{J}C_{e}}\right)\;\;\;\;\;\;\;\;\;\;\;\;\;\;\;\;\;\;\;\;\;\;\;\;\;\;\;\left(10\right)$
Thermodynamics	Van't Hoff	$\begin{array}{c} \ln\left(K_{c}\right)=\frac{\Delta S^{o}}{R}-\frac{\Delta H^{o}}{RT}\;\;\;\;\;\;\;\;\;\;\;\;\;\;\;\;\;\;\;\;\left(11\right)\\ K_{c}=\frac{q_{e}}{c_{e}}\;and\;\Delta G^{o}=-RT\times \ln\left(K_{c}\right) \end{array}$

The thermodynamic parameters of fluoride adsorption in aqueous media were also evaluated using a thermodynamic model known as the van't Hoff model, which was commonly used to automatically identify the spontaneity of the adsorption process.^[^ ^50^ ^]^ The CAVS adsorption evaluation software was used to evaluate all these models to determine that one fits best.

q_t_ was adsorption over time (mg g^−1^), q_e_ was adsorption at equilibrium (mg g^−1^), C was a constant of any experiment (mg g^−1^), k_1_ was the PFO rate constant (min^−1^), k_2_ was the PSO rate constant (g mg^−1^ min^−1^), k_2_q_e_^2^ was the initial PSO adsorption rate (mg g^−1^ min^−1^), Ce was the aqueous concentration at equilibrium (mg L^−1^), K.F. reflects the adsorbent capacity (L g^−1^), a_F_ was Freundlich isotherm constant that the heterogeneity factor (unitless) ranging from 0 to 1, K.L. reflects the solute adsorptivity (L g^−1^), b_L_ (L mg^−1^) was Langmuir isotherm constant related to the energy of adsorption and K.L./b_L_ was defined as the monolayer adsorbent capacity, b_T_ was the Temkin isotherm regular associated with the heat of adsorption and A_T_ (L g^−1^) was the Temkin isotherm equilibrium binding constant, q_m_ was the theoretical adsorption isotherm saturation capacity (mg g^−1^), K.J. was the Jovanovic isotherm constant (L g^−1^), K_c_ was the linear adsorption distribution coefficient or single point (dimensionless), ΔS^o^ (J mol^−1^) was change in enthropy, ΔH^o^ (K J mol^−1^) was change in enthalpy, R was universal gas constant, T (°C) was temperature, ΔG^o^ (K J mol^−1^) was change in Gibb's free energy.

## Conflict of Interest

The authors declare no conflict of interest.

## Data Availability

The data that support the findings of this study are available from the corresponding author upon reasonable request.
